# Diffuse alveolar hemorrhage after use of a fluoropolymer-based waterproofing spray

**DOI:** 10.1186/s40064-015-1079-3

**Published:** 2015-06-17

**Authors:** Ryota Kikuchi, Masayuki Itoh, Tomonori Uruma, Takao Tsuji, Hidehiro Watanabe, Hiroyuki Nakamura, Kazutetsu Aoshiba

**Affiliations:** Department of Respiratory Medicine, Tokyo Medical University Ibaraki Medical Center, 3-20-1 Chuou, Ami, Inashiki, Ibaraki 300-0395 Japan

**Keywords:** Alveolar hemorrhage, Waterproofing spray, Fluoropolymer, Respiratory insufficiency, Dyspnea

## Abstract

A 30-year-old man developed chills, cough and dyspnea a few minutes after using a fluoropolymer-based waterproofing spray in a small closed room. He visited our hospital 1 h later. Examination revealed that the patient had incessant cough, tachypnea, fever and decreased peripheral arterial oxygen saturation. Blood tests revealed leukocytosis with elevated serum C-reactive protein levels. Chest radiographs and computed tomography (CT) scan showed bilateral ground glass opacities, mainly in the upper lobes. Bronchoalveolar lavage (BAL) fluid obtained from the right middle lobe showed a bloody appearance. Microscopic examination of a BAL cytospin specimen revealed the presence of numerous red blood cells associated with extreme neutrophilia. Microbiological studies of the BAL fluid were negative. The patient was observed without corticosteroid therapy, and his symptoms and abnormal shadows on the chest radiographs and CT improved. On day 7 after admission, the patient was discharged from the hospital. Accidental inhalation of waterproofing spray may cause diffuse alveolar hemorrhage, a rare manifestation of acute lung injury. Supportive treatment may be effective and sufficient.

## Case presentation

**Figure 1:**
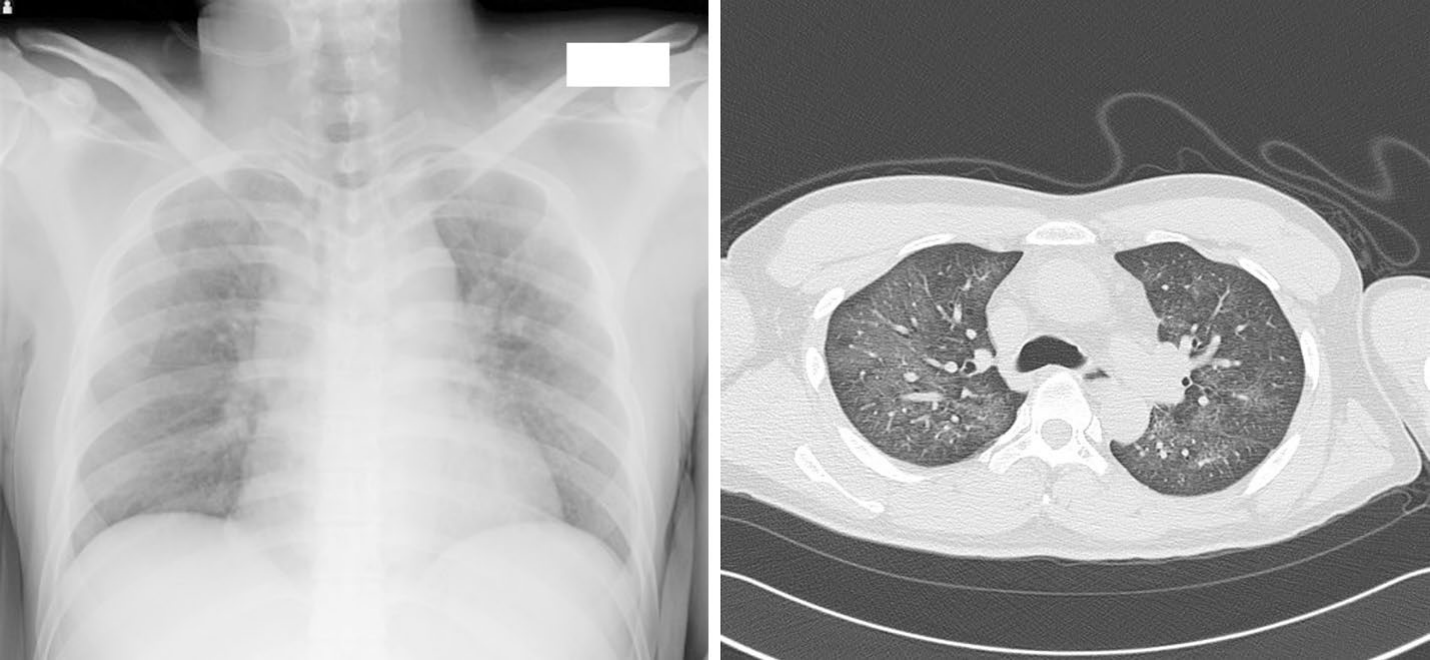
A radiograph (*left*) and high resolution CT scan (*right*) of the chest showing bilateral ground glass opacities.

**Figure 2:**
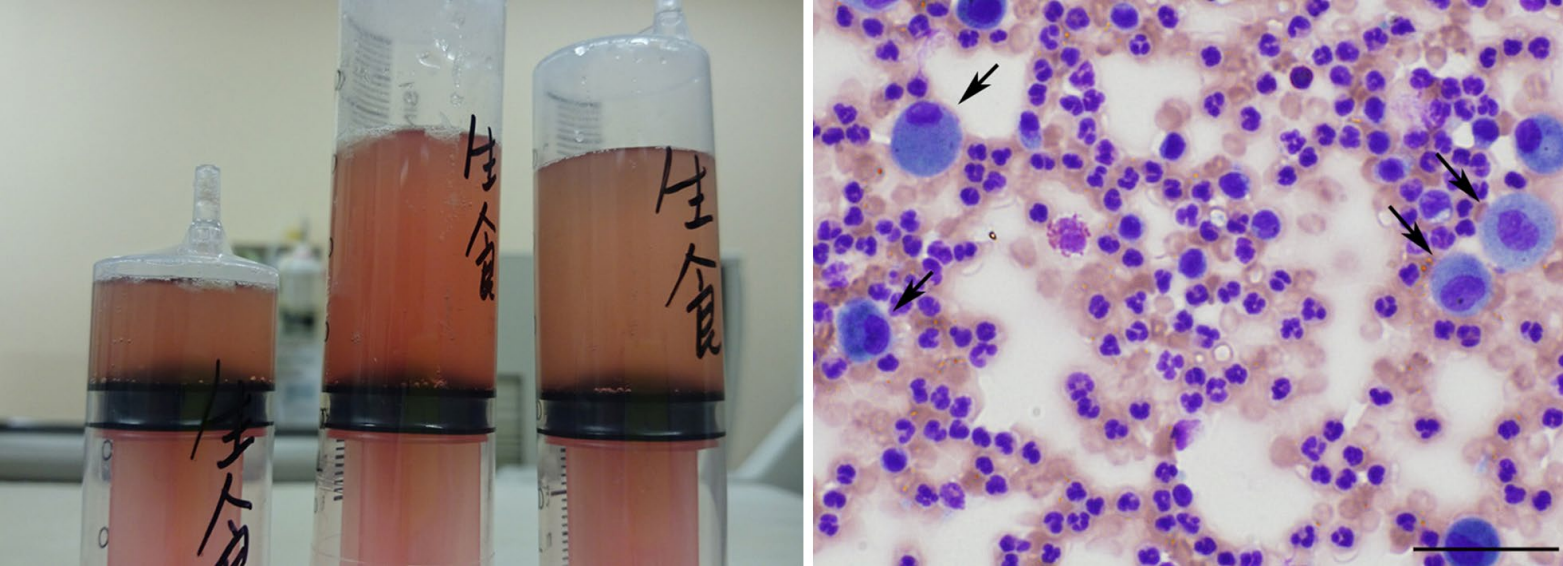
Bloody gross appearance (*left*) and microscopic findings (*right*) of bronchoalveolar lavage fluid specimens. *Right* May-Giemsa-stained cytospin preparation showing numerous red blood cells and neutrophils, consistent with the diagnosis of alveolar hemorrhage. *Arrows* macrophages. *Scale bar* 50 μm.

A 30-year-old Japanese male, a smoker with no significant past medical, allergy or cocaine use history, developed chills, cough and dyspnea a few minutes after using a fluoropolymer-based waterproofing spray on his leather uniforms in a small closed room. He presented to our hospital 1 h later. Examination revealed that the patient had incessant cough, tachypnea (30 breaths per minute), fever (39.4°C) and slightly decreased peripheral arterial oxygen saturation [percutaneous oxygen saturation (SpO_2_): 92% on room air]; however, there were no lung crackles or wheeze. Examination of the cardiovascular system revealed no abnormalities. There was no weight loss, arthralgia or skin rash. As shown in Table 1, the blood tests revealed leukocytosis [20,300 cells/μL (normal, 4,000–8,000 cells/μL) with 91.5% neutrophils, 0% eosinophils and 6.5% lymphocytes], elevated serum C-reactive protein levels [3.14 mg/dL (normal, <0.3 mg/dL)] and elevated serum LDH levels [341 U/l (normal, 106–220 U/l)]. Autoimmune screening, including for anti-neutrophil cytoplasm antibody (ANCA) and anti-glomerular basement membrane (anti-GBM) antibody, revealed no autoantibodies. The serum brain natriuretic peptide (BNP) level and coagulation profile were normal. Urinalysis showed no proteinuria or hematuria. Chest radiographs and computed tomography (CT) scan showed bilateral ground glass opacities, mainly in the upper lobes (Figure 1). Fiberoptic bronchoscopy was performed 13 h after the onset of the symptoms. Bronchoalveolar lavage (BAL) fluid obtained from the right middle lobe showed a bloody appearance (Figure 2, left). While the upper lobes appeared to be predominantly involved, the middle lobe was selected for obtaining the BAL sample, because this lobe is the smallest and yields the largest return (Baughman 2007). Microscopic examination of a BAL cytospin specimen revealed the presence of numerous red blood cells with extreme neutrophilia (differential neutrophil count 79.8%, normal range <3%) (Figure 2, right). The siderophage count was less than 2%. Microbiological studies of the BAL fluid were negative. The patient was observed without corticosteroid therapy, and his symptoms improved significantly by the day after admission. By day 7 after admission, the abnormal shadows on the chest radiographs and CT scan had almost completely disappeared and the patient was discharged from the hospital. At 6 months after discharge, his health status was normal. Consent to publish this case report was obtained from the patient.

**Table 1:** Laboratory data on admission

Hematology	
White blood cells	20,300/μl (H)
Neutrophils	91.5% (H)
Eosinophils	0.0%
Basophils	0.0%
Lymphocytes	6.5%
Monocytes	2.0%
Hemoglobin	16.5 g/dl
Platelets	273,000/μl
Biochemistry	
Total protein	7.0 g/dl
Albumin	4.6 g/dl
Blood urea nitrogen	11.5 mg/dl
Creatinine	0.97 mg/dl
Lactate dehydrogenase	341 U/l (H)
Aspartate aminotransferase	26 U/l
Alanine aminotransferase	12 U/l
Coagulation	
PT-INR	1.06
APTT	29.5 s
APTT (control)	34.0 s
Serology	
C-reactive protein	3.14 mg/dl (H)
KL-6	339 U/ml
Surfactant protein-D	17.2 ng/ml
B-D-glucan	<6.0 pg/ml
Brain natriuretic peptide	10.2 pg/ml
Anti-GBM antibody	<10 U/ml
Antinuclear antibody	<×40
Anti-ds-DNA antibody	<1.2 IU/ml
Anti-SS-A antibody	<1.0 U/ml
Anti-SS-B antibody	<1.0 U/ml
Anti-Scl-70 antibody	<1.0 U/ml
Anti-Jo-1 antibody	−
MPO-ANCA	<1.0 U/ml
PR3-ANCA	<1.0 U/ml
Urinalysis	
Protein	−
Sugar	−
Occult blood	–
Bronchoalveolar lavage (rt.B^4^a)	
Recovery rate	53.3% (80/150 ml)
Cell count	1.2 × 10^6^/μl
Macrophages	20.0%
Lymphocytes	0.8%
Eosinophils	0.4%
Neutrophils	78.8% (H)
Siderophages	1.9%
Culture	
Bacteria	−
Mycobacterium tuberculosis	−
Cytology	Class II

*PT-INR* prothrombin time-international normalized ratio, *APTT* activated partial thromboplastin time, *KL-6* sialylated carbohydrate antigen KL-6, *anti-GBM antibody* anti-anti-glomerular basement membrane antibody, *anti-ds-DNA antibody* anti-double stranded DNA, *anti-SS-A antibody* anti-Sjögren’s-syndrome-related antigen A antibody, *anti-SS-B antibody* anti-Sjögren’s-syndrome-related antigen B antibody, *anti-Scl-70 antibody* anti-scleroderma-70 antibody, *anti-Jo-1 antibody* anti-histidyl tRNA synthetase antibody, *MPO-ANCA* myeloperoxidase-antineutrophil cytoplasmic antibody, *PR3-ANCA* proteinase 3- antineutrophil cytoplasmic antibody, *H* indicates high, *−* indicates negative.

## Discussion

Accidental inhalation of waterproofing spray has been reported to cause lung injury (Vernez et al. 2006). Diffuse alveolar hemorrhage (DAH) is a rare, but serious manifestation of acute lung injury (Fukui et al. 2011). DAH in this patient was unlikely to be due to immune causes such as microscopic polyangiitis, systemic lupus erythematosus or Goodpasture’s syndrome, because of the absence of weight loss, arthralgia, proteinuria, hematuria, anti-nuclear antibody, anti–anti-GBM antibody or ANCA (Picard et al. 2010). Furthermore, neither congestive heart failure nor coagulopathy could be considered as the likely cause of the DAH, because the serum BNP level and coagulation profile were normal.

The toxicity of waterproofing sprays is thought to be due to their content of water-repellents, such as fluoropolymers and silicon polymers, or of the solvent in which they are delivered (Picard et al. 2010). Among different repelling agents, fluoropolymers have been the most studied with regard to their toxicity following inhalation (Hays and Spiller 2014). An animal study in mice demonstrated that inhalation of fluoropolymers resulted in pulmonary collapse and pneumonia, probably by counteracting the surfactant action in the alveoli of the lung, whereas mice exposed to non-fluoropolymer-containing products showed less severe injury (Yamashita and Tanaka 1995). These findings suggest that the fluoropolymer water repellents in waterproofing sprays are responsible for causing the acute respiratory illness following inhalation. The primary structural changes after inhalation of waterproofing sprays in animals include alveolar type I cell necrosis and alveolar type II cell necrosis, with resultant impairment of surfactant secretion, direct counteraction of surfactant action, alveolar atelectasis and hemorrhage (Hays and Spiller 2014). A recent study reported developing an isolated perfused rat lung model to examine the potential of surface tension-active substances in waterproofing sprays to cause pulmonary collapse (Fischer et al. 2012). Other mechanisms of the toxicity of waterproofing sprays include an indirect and complex mechanism requiring metabolic activation with or without interaction with other factors, such as solvents (e.g., n-heptane, hexane and petroleum distillates) and smoking (Hays and Spiller 2014). The mist particle size and the emission rate have also been shown to influence the toxicity of waterproofing sprays (Hays and Spiller 2014; Yamashita et al. 1997).

The commonest symptoms associated with inhalation of fluoropolymer-based waterproofing sprays include non-productive cough and dyspnea, often accompanied by flu-like symptoms (Vernez et al. 2006; Hays and Spiller 2014). The illness develops rapidly, usually within minutes to hours (Vernez et al. 2006; Hays and Spiller 2014). Similar to the case reported herein, there is one previous report of DAH resulting from inhalation of a waterproofing spray (Fukui et al. 2011). Laboratory studies generally reveal evidence of acute inflammation such as leukocytosis and elevated serum CRP (Hays and Spiller 2014). In one previous case report, marked hypocalcemia was observed in the patient, which was attributed to the binding activity of fluoride to cations, such as calcium (Bracco and Favre 1998). Serious outcomes and death are uncommon (Vernez et al. 2006; Hays and Spiller 2014). Most victims, even those that present DAH, like our patient, improved with supportive care, with or without corticosteroid and inhaled β_2_ adrenergic agonist therapy (Vernez et al. 2006; Fukui et al. 2011; Hays and Spiller 2014).

## Conclusions

Accidental inhalation of waterproofing sprays may cause diffuse alveolar hemorrhage, a rare manifestation of acute lung injury. Supportive treatment may be effective and sufficient.

## References

[CR1] Baughman RP (2007). Technical aspects of bronchoalveolar lavage: recommendations for a standard procedure. Semin Respir Crit Care Med.

[CR2] Bracco D, Favre JB (1998). Pulmonary injury after ski wax inhalation exposure. Ann Emerg Med.

[CR3] Fischer M, Koch W, Windt H, Dasenbrock C (2012). A pilot study on the treatment of acute inhalation toxicity studies: the isolated perfusion rat lung as a screening tool for surface-active substances. Altern Lab Anim.

[CR4] Fukui Y, Tanino Y, Doshita K, Nakano H, Okamoto Y (2011). Diffuse alveolar hemorrhage arising after use of a waterproofing spray. Nihon Kokyuki Gakkai Zasshi.

[CR5] Hays HL, Spiller H (2014). Fluoropolymer-associated illness. Clin Toxicol.

[CR6] Picard C, Cadranel J, Porcher R, Prigent H, Levy P, Fartoukh M (2010). Alveolar haemorrhage in the immunocompetent host: a scale for early diagnosis of an immune cause. Respiration.

[CR7] Vernez D, Bruzzi R, Kupferschmidt H, De-Batz Droz P, Lazor R (2006). Acute respiratory syndrome after inhalation of waterproofing sprays: a posteriori exposure-response assessment in 102 cases. J Occup Environ Hyg.

[CR8] Yamashita M, Tanaka J (1995). Pulmonary collapse and pneumonia due to inhalation of a waterproofing aerosol in female CD-1 mice. J Toxicol Clin Toxicol.

[CR9] Yamashita M, Yamashita M, Tanaka J, Hirai H, Suzuki M, Kajigaya H (1997). Toxicity of waterproofing spray is influenced by the mist particle size. Vet Hum Toxicol.

